# Brain structural alterations associated with young women with subthreshold depression

**DOI:** 10.1038/srep09707

**Published:** 2015-05-18

**Authors:** Haijiang Li, Dongtao Wei, Jiangzhou Sun, Qunlin Chen, Qinglin Zhang, Jiang Qiu

**Affiliations:** 1 Key Laboratory of Cognition and Personality (SWU), Ministry of Education, Chongqing 400715, China; 2 Faculty of Psychology, Southwest University, Chongqing 400715, China

## Abstract

Neuroanatomical abnormalities in patients with major depression disorder (MDD) have been attracted great research attention. However, the structural alterations associated with subthreshold depression (StD) remain unclear and, therefore, require further investigation. In this study, 42 young women with StD, and 30 matched non-depressed controls (NCs) were identified based on two-time Beck Depression Inventory scores. Whole-brain voxel-based morphometry (VBM) and region of interest method were used to investigate altered gray matter volume (GMV) and white matter volume (WMV) among a non-clinical sample of young women with StD. VBM results indicated that young women with StD showed significantly decreased GMV in the right inferior parietal lobule than NCs; increased GMV in the amygdala, posterior cingulate cortex, and precuneus; and increased WMV in the posterior cingulate cortex and precuneus. Together, structural alterations in specific brain regions, which are known to be involved in the fronto-limbic circuits implicated in depression may precede the occurrence of depressive episodes and influence the development of MDD.

Depression is one of the most common mental disorders in the adult population^1^. Subthreshold depression (StD), defined as subjects with scores above a cut-off score in a self-report depressive measure but do not fulfill the criteria for major depression disorder (MDD), is more prevalent than MDD^2^. Studies found that StD has a serious effect on the quality of life and daily functioning and also increases societal medical burden^3^,^4^.

Brain structural imaging has been widely used to explore structures and associated circuits involved in the pathophysiology of MDD, including prefrontal cortex (PFC), anterior cingulate cortex (ACC), hippocampus, and amygdala^5^,^6^,^7^. Individuals with major depression have reduced gray matter volume (GMV) in the medial PFC and ACC when compared with non-depressed controls (NCs)^8^. In addition, decreased hippocampus and amygdala volumes in depressed individuals have been reported as well^9^,^10^.

Structural alterations in similar regions have also been reported in populations with elevated risk for depression or with StD. Although the evidence base is similar, the direction of effects sometimes differs from that observed in MDD^1^,^11^. For instance, previous studies reported larger amygdala volume in participants with elevated risk for depression^12^,^13^, whereas others observed no volumetric differences in the amygdala^14^. Findings regarding the hippocampus also showed inconsistent results. Spalletta and colleagues^15^ found reduced hippocampus volume only in healthy males with subclinical depression. Meanwhile, such results were not observed in other studies that recruited only women with subclinical depression^16^. In addition, studies involving participants with elevated risk for depression mainly reported a smaller volume in the PFC, such as mPFC and ACC, compared with NCs^1^,^11^,^14^, but examination of cortical thickness in adolescents with elevated risk for depression showed increased thickness in some regions of PFC, such as mPFC and ACC^17^.

Previous research mainly examined structural abnormalities in MDD. The brain structural abnormalities concerning StD remain unclear and, thus, require further investigation. Considering that StD is regarded as the prodromal phase of MDD^2^,^3^ and can predict the occurrence of depressive disorders^18^, it is important to gain a better understanding of this disorder. Although previous studies have investigated the structural variances in individuals with StD or with high risk for depression^14^,^15^,^16^, the results are inconsistent and significantly differ from those of the current study in several aspects. First, unlike previous studies which used adolescents^19^ or elderly^20^ samples, the present study focuses on exploring structural variances among young adults (ages 18 to 24 years) with StD, because this developmental period (ages 18 to 24 years) is a critical transition period^21^ characterized by higher than average stress levels compared with other developmental phases^22^. Moreover, age-related alterations in brain structure are ongoing during young adulthood^23^, especially in the PFC, a region crucial for self-control and emotional regulation^24^. Delayed maturation of brain structures and multiple stressors may also increase risk for mood disorders among young adults^25^. Second, to recruit individuals that are truly under the state of StD, we used a relatively more standard two-time screening procedure employing the commonly used Beck Depression Inventory, which has not been utilized by the aforementioned studies. Third, inconsistent findings observed in previous studies may be due to the usage of different analytic strategies (region of interest, ROI or whole-brain survey). To obtain a clearer picture of structural variances associated with StD, the current study combines both ROI and whole-brain analysis.

In summary, the goal of the current research is to explore structural alterations associated with StD in a sample of non-clinical, healthy young women using voxel-based morphometry (VBM). Based on the findings of previous structural imaging studies with psychiatric and high risk samples, we posit that young women with StD would have associated structural abnormalities in the fronto-limbic brain circuits including the regions of mPFC, ACC, hippocampus and amygdala.

## Results

### Behavioral data

Characteristics for participants in the StD and NC groups are presented in Table 1. Participants in the StD and NC groups did not differ in age, *t*_(70)_ = 0.24, *p* = 0.81; Combined Raven's Test (CRT) score, *t*_(70)_ = −1.33, *p* = 0.19; global gray matter volume, *t*_(70)_ = 1.00, *p* = 0.32; or global white matter volume, *t*_(70)_ = 1.46, *p* = 0.15. However, they differed in Beck Depression Inventory (BDI) scores, *t*_(70)_ = 16.80, *p* < 0.0001.

### Regional GMV alterations in StD - Whole brain analysis

**Figure 1:**
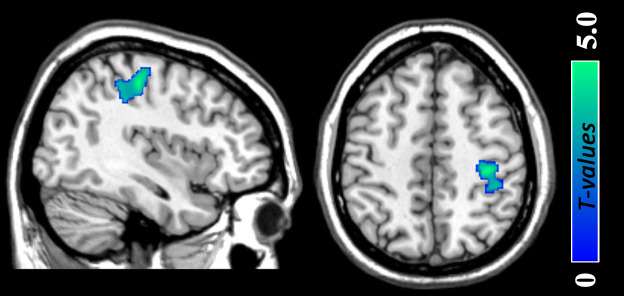
Gray matter volume reductions in the regions of right inferior parietal lobule and postcentral gyrus (peak MNI coordinate: 36, −25, 49; *t* = 4.91, *p (corrected)* < 0.05) among young women with subthreshold depression as compared with non-depressed controls. The color density represents the T score.

**Figure 2:**
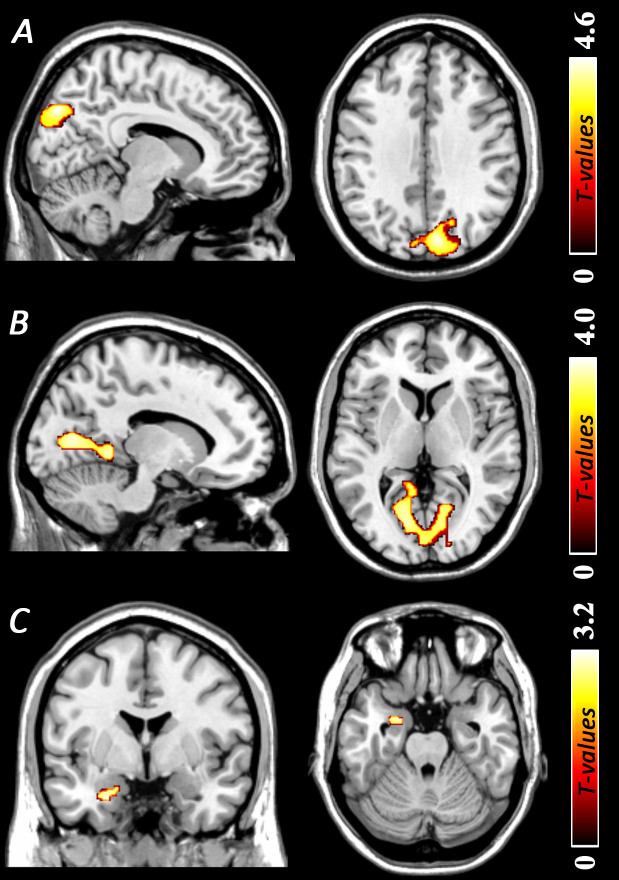
Regions of gray matter volume increases among young women with subthreshold depression as compared with non-depressed controls. (A) Gray matter volume increases in the regions of bilateral precuneus, cuneus and superior occipital gyrus (peak MNI coordinate: 9, −79, 36; *t* = 4.57, *p (corrected)* < 0.05). (B) Gray matter volume increases in the regions of bilateral posterior cingulate cortex, calcarine and lingual gyrus (peak MNI coordinate: −18, −72, 12; *t* = 3.99, *p* *(corrected)* < 0.05). (C) Gray matter volume increases in the left amygdala (peak MNI coordinate: −26, 2, −27; *t* = 3.28, *p*_(SVC)_ < 0.05). The color density represents the T score. SVC, small volume correction.

Compared with NCs, participants with StD showed significantly decreased GMV in a cluster that mainly included regions of the right inferior parietal lobule (IPL) and postcentral gyrus (peak MNI coordinate: 36, −25, 49; *t* = 4.91, *p (corrected)* < 0.05; *Cluster size* = 2585 mm^3^; Fig. 1; Table 2). Furthermore, participants with StD showed significantly increased GMV (1) in a cluster that mainly included the regions of bilateral precuneus (PreC), cuneus and superior occipital gyrus (peak MNI coordinate: 9, −79, 36; *t* = 4.57, *p (corrected)* < 0.05; *Cluster size* = 5072 mm^3^; Fig. 2A; Table 2); and (2) in a cluster that mainly included the regions of bilateral posterior cingulate cortex (PCC), calcarine and lingual gyrus (peak MNI coordinate: −18, −72, 12; *t* = 3.99, *p* *(corrected)* = 0.054; *Cluster size* = 9085 mm^3^; Fig. 2B; Table 2).

### Regional GMV alterations in StD - ROI based analyses

To examine whether there were any differences in GMV between StD and NC groups in *a priori* regions, we conducted separate analyses using predefined ROI. Results indicated that individuals with StD had increased GMV in the left amygdala (peak MNI coordinate: −26, 2, −27; *t* = 3.28, *p*_(SVC)_ < 0.05; *Cluster size* = 310 mm^3^; Fig. 2C; Table 2) as compared with NCs. No other significant effects were found.

### Regional WMV alterations in StD

**Figure 3:**
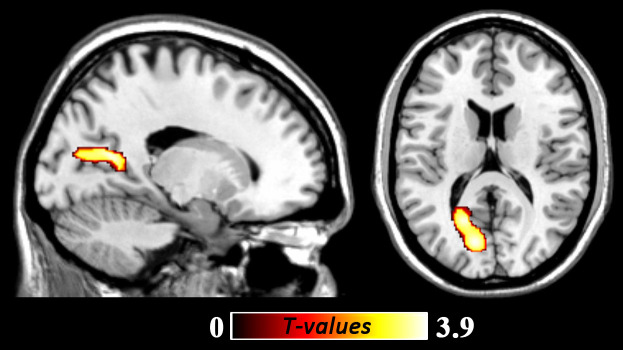
White matter volume increases in the regions of left posterior cingulate cortex, precuneus, and calcarine (peak MNI coordinate: −12, −76, 15; *t* = 3.86, *p* *(corrected)* < 0.05) among young women with subthreshold depression as compared with non-depressed controls. The color density represents the T score.

Compared to NCs, participants with StD showed significantly increased white matter volume (WMV) in a cluster that mainly included the regions of left PCC, PreC and calcarine (peak MNI coordinate: −12, −76, 15; *t* = 3.86, *p (corrected)* < 0.05; *Cluster size* = 4208 mm^3^; Fig. 3; Table 2). No other significant effects were found.

## Discussion

The goal of the present study is to characterize brain structural alterations associated with StD in a sample of non-clinical, healthy young women using VBM. Structural alterations were observed in GMV and WMV in participants with StD. The main findings were that, compared with NCs, participants with StD had decreased GMV in the right inferior parietal lobule; and increased GMV in the left amygdala and bilateral precuneus (PreC) and posterior cingulate cortex (PCC). In addition, compared with NCs, participants with StD had increased WMV in the PCC and PreC. These results provide direct neuroanatomical evidence for structural alterations associated with depressive symptoms in young female adults, even at a subthreshold level.

Consistent with our hypothesis, amygdala volume alterations were observed in young women with StD. Unlike reports of decreased GMV of amygdala in patients with MDD^5^,^26^, the present study observed increased GMV of amygdala among young women with StD as compared with NCs. In fact, the amygdala findings are highly inconsistent among studies on depression^6^. However, most structural imaging studies on populations at risk for depression found greater amygdala volume than NCs^12^,^13^, which is in line with our findings. In addition, recent studies have found the association between increased amygdala volume and heightened negative affect^27^ and negative memory bias^28^, which are regarded as psychological characteristics and vulnerability factors of depression.

Decreased GMV in right anterior IPL and increased GMV in PCC and PreC were also observed among young women with StD, although these regions were not included in our *a priori* hypotheses. As a core component of the frontoparietal control system^29^, the anterior IPL has been implicated in cognitive control, episodic memory^30^,^44^, and integration of perceptual information and detection of conflict^29^. The dysfunction of the anterior IPL in depression has been connected to deficits in emotion processing, audiovisual integration, and impaired memory^31^. Reduced IPL volume or cortical thickness have been observed among individuals with elevated depressive symptoms^32^, familial risk for major depression disorder^17^, and untreated first episode MDD^33^. Thus, the reduced IPL volume may induce inefficient attentional control on negative emotion processing. In relation to this, previous studies have documented that biased processing of negative information contributes to the onset and maintenance of the depression^34^.

In this study, we also found that GMV of the PCC and PreC were significantly increased in young women with StD. The role of PCC and PreC in emotion evaluation and episodic memory has been described in functional neuroimaging studies, which reported hyperactivity in the PCC and PreC in evaluating emotional stimuli^35^. Recent structural imaging study reported increased gray matter density in the regions of PCC and PreC among bipolar patients as compared with NCs^36^. Significantly positive correlation between depressive symptoms and PCC volume have also been observed^37^. Thus, increased PCC/PreC GMV may be correlated with deficits in emotion evaluation and episodic memory retrieval bias in StD.

Greater regional WMV in the regions of PCC and PreC in young women with StD may reflect abnormal fibers connections that influence neural transmission in the area and among networks. The PCC and PreC regions interact with the medial PFC composed a distributed network during rest, default mode network (DMN)^38^. DMN has been found to be overactive among depressive disorders both during rest^39^ and when facing emotional stimuli^40^, which is associated with increased negative rumination in depressive disorders^39^. Thus, enhanced WMV in PCC and PreC may affect the functional interactions between PreC/PCC and other regions among DMN in young women with StD.

We found no volumetric alteration in the ACC and mPFC in young women with StD, although brain volume alterations has been reported in patients with MDD. In fact, volume changes in the regions of PFC among individuals with high risk for depression is controversial^12^,^41^,^42^. Taki and colleagues found reduced GMV in the bilateral mPFC in male individuals with StD^41^. However, researcher also reported no significant alteration in PFC volume^42^ or greater GMV in PFC^12^ among individuals with high risk for depression. These inconsistent results may due to the difference in study samples, depression measures employing and participants screening methods^14^. The inconsistency also underlines that more evidence is needed in future studies to explore structural variance associated with subthreshold depression.

Notwithstanding its potential implications, the main limitations of this study should be acknowledged. First, only young women were recruited in the present study, which may limit the generalizability of the findings to men. However, previous studies reported that both depression and anxiety disorders are more prevalent among women than among men^43^. In addition, little is known about possible gender differences in brain structure in StD^15^, thus, we decided to choose only female participants to reduce the heterogeneity of our sample. Future research could investigate gender differences in the structural alteration among individuals with StD.

Second, this study was conducted in a sample with StD, and, thus caution should be warranted in making any conclusions about clinical symptoms levels of depression. Third, whereas limiting the assessment to young adults was advantageous in examining structural alterations between individuals with StD and NCs independent of aging and life experience, it remains unclear whether the current findings are generalizable to older cohorts, in which potentially unique relations between depression and structural changes may be present. For example, developmental studies have reported age-related decreases in GMV and WMV, particularly between middle and late adulthood, and found links to cumulative effects of stress and glucocorticoids^44^. Consequently, future research on structural alterations between individuals with StD and NCs is needed, particularly in non-clinical samples over the age of 50 years.

In conclusion, this study explored structural alterations in both GMV and WMV in a non-clinical sample of young women with StD using VBM. The findings show that altered GMV and WMV in the amygdala, IPL, PCC, and PreC may be structural markers of young adults with StD. Thus, the current study demonstrates a unique structural abnormality in young women with StD, which is distributed across distinct gray and white matter areas of the brain.

## Methods

### Participants

A total of 72 female undergraduate students were recruited from Southwest University, Chongqing, China. The specific steps in selecting the participants are described here. First, participants completed the BDI^45^ during the mass pre-testing. Participants who scored 14 and above or 6 and below were invited to participate in a second session approximately 1 week later. In this session, potential participants completed an in-person screening session, which included BDI and administration of the Structured Clinical Interview for DSM-IV-TR Axis I Disorders (SCID)^46^. The inclusion criteria included the following: (1) didn't fulfil the SCID diagnostic criteria for MDD; (2) had no current bipolar disorder, panic disorder or schizophrenia; (3) had no concurrent psychotherapy and psychotropic medication; and (4) not pregnancy and currently not in their menstrual period.

Finally, 46 subjects with a BDI score of 14 and above at second points in time were assigned to the StD group, whereas 33 subjects with a BDI score of 6 and below at second points in time were assigned to non-depressed controls (NCs). After checking structural imaging data, four participants in the StD group and three participants in the NC group were excluded from the analyses due to movement artifacts or incomplete brain scans. Therefore, the present study involved 42 subjects in the StD group and 30 subjects in the NC group in all analyses. All participants were right-handed and had no life history of neurological injury or disease. The study was approved by the SWU Brain Imaging Center Institutional Review Board.

In accordance with the Declaration of Helsinki (2008), written informed consent was obtained prior to engagement in the research tasks. First, all participants completed Combined Raven's Test (CRT)^47^ and other measures not report here. Then, participants underwent an MRI scan wherein they were instructed to keep their heads still and to remain awake. The scan was comprised of anatomical imaging, resting state imaging and other task related functional imaging, only anatomical imaging data was used in this study. After completing all study protocols, participants were thanked for their time and received financial compensation.

### Measures

#### Assessment of general intelligence

To control for individual differences in intellectual ability in analyses of regional GMV and WMV difference between StD and NCs^48^, participants completed the CRT, a recognized intelligence test with a high degree of reliability and validity. The CRT, which includes the Raven's standard progressive matrices (C, D, E sets) and Raven's colored progressive matrices (A, B, AB sets), consists of 72 items revised for use in Chinese samples by Sun et al.^47^. Number of correct answers given in 40 minutes was used as a psychometric index of individual intelligence in line with standard practice^49^.

#### Beck depression inventory-II

The BDI is a 21 item self-report questionnaire measuring severity of depressive symptoms during the past week on four-point scale (0–3), with higher scores indicating more severe symptomatology^45^. The BDI is considered to be a valid and reliable measure assessing the severity of depressive symptom in clinical and non-clinical samples^50^.

#### Image acquisition

MR images were acquired on a 3.0-T Siemens Trio MRI scanner with an 8-channel head coil (Siemens Medical, Erlangen, Germany). High-resolution T1-weighted anatomical images were acquired using a magnetization-prepared rapid gradient echo (MPRAGE) sequence (TR/TE/TI = 1900 ms/2.52 ms/900 ms; Flip angle = 9°; Slices = 176; Slice thickness = 1.0 mm; Resolution matrix = 256 × 256; Voxel size = 1 × 1 × 1 mm^3^).

#### Image processing

Images were processed using the SPM8 (Wellcome Department of Cognitive Neurology, London, UK) implemented in Matlab 7.8 (MathWorks Inc., Natick, MA, USA). Each MR image was first displayed in SPM8 to screen for artifacts or gross anatomical abnormalities. For better registration, the re-orientation of images was manually set to the anterior commissure. The T1 weighted anatomical images underwent an initial segmentation process in SPM8 using new segmentation which simultaneously estimated the transformation parameters for warping grey matter (GM) and white matter (WM) tissue probability maps (TPMs) onto the images. After affine registration, the GM and WM segments of all 72 participants in both groups were processed to create study-specific templates using DARTEL algorithm^51^. The native space GM and WM segments, DARTEL template, and flow fields from the template creation were normalized to Montreal Neurological Institute (MNI) standard space and resampled to an isotropic resolution of 1.5 mm. To preserve the original GM and WM volumes, the image intensity of each voxel was modulated by Jacobian determinants. Finally, normalized, modulated and resampled images (GM and WM images) were smoothed with a 10-mm full-width at half-maximum (FWHM) Gaussian kernel to increase signal to noise ratio.

#### Statistical analysis

Statistical analyses of GMV and WMV data were performed using SPM8. In the whole-brain analyses, we performed a two sample t-test in the SPM8 to determine whether there were any significant regional GMV or WMV differences between StD and NCs. To control for possible confounding variables, age, CRT and global volumes of GM or WM were entered as covariates. To avoid edge effects around the borders between GM and WM, an absolute threshold masking of 0.2 was used; that is, voxels with GM or WM values lower than 0.2 were excluded from analyses^52^,^53^.

For the *a priori* regions of interest (mPFC, ACC, amygdala, and hippocampus), statistical significance level was set at P < 0.05 with small volume correction (SVC)^54^ for multiple comparisons in the regions of interests (ROI). The amygdala, ACC and hippocampus masks were based on WFU PickAtlas^55^. For mPFC is not clearly defined anatomically in WFU PickAtlas, we utilized bilateral superior medial frontal gyrus based on the anatomic labels specified in the WFU PickAtlas to comprise a mPFC ROI mask following previous study^56^.

For regions not specified *a priori*, a threshold of P < 0.05 corrected for the whole-brain volume at a cluster level using non-stationary correction^57^ with an underlying voxel level of *p* < 0.0025 were used as suggested by previous study^58^.

## Author Contributions

H.J.L. collected, analyzed, interpreted the data, and wrote the paper. D.T.W., Q.L.C. and J.Z.S helped to collected, analyzed, interpreted the data. J.Q. and Q.L.Z. interpreted the data, and help to wrote the paper.

## Additional Information

**How to cite this article**: Li, H. *et al.* Brain structural alterations associated with young women with subthreshold depression. *Sci. Rep.* 5, 9707; DOI:10.1038/srep09707 (2015).

## Figures and Tables

**Table 1:** Characteristics for young women in subthreshold depression and non-depressed controls

	Subthreshold depression (42)		Non-depressed controls (30)		*T*-test	
	*Mean*	*SD*	*Mean*	*SD*	*t score*	*p*
Age	20.26	0.89	20.20	1.30	0.24	0.81
CRT	63.29	6.26	65.03	4.23	1.33	0.19
BDI	22.74	5.88	3.87	2.11	16.80	0.0001
Global GMV	0.39	0.03	0.38	0.02	1.00	0.32
Global WMV	0.44	0.03	0.43	0.02	1.46	0.15

Note: CRT: Combined Raven's Test; BDI, Beck Depression Inventory II; GMV, gray matter volume; WMV, white matter volume.

**Table 2:** Differences in gray-matter and white-matter volumes between young women with subthreshold depression and non-depressed controls

	Brain regions		MNI coordination			Cluster size (mm^3^)	Peak T-Value
			x	y	z		
Altered GMV in StD							
	*Decreased GMV*						
	IPL/Postcentral gyrus	R	36	−25	49	2585	4.91
	*Increased GMV*						
	Precuneus/Cuneus	R/L	9	−79	36	5072	4.57
	PCC/Calcarine/Lingual gyrus	R/L	−18	−72	12	9085	3.99
	Amygdala	R	−26	2	−27	310	3.28*
Altered WMV in StD							
	*Increased GMV*						
	PCC/Precuneus/Calcarine	L	−12	−76	15	4208	3.86

Note: GMV, gray matter volume; WMV, white matter volume; StD, subthreshold depression; IPL, inferior parietal lobule; PCC, posterior cingulate cortex.

*result significant at *p* < 0.05 using the small volume correction.

## References

[b1] Gotlib I. H., Joormann J. & Foland-Ross L. C. Understanding familial risk for depression: A 25-year perspective. Perspect. Psychol. Sci. 9, 94–108 (2014). 10.1177/1745691613513469 PMC11877285 26173248

[b2] Cuijpers P., Smit F. & Van Straten A. Psychological treatments of subthreshold depression: a meta-analytic review. Acta. Psychiat. Scand. 115, 434–441 (2007). 17498154 10.1111/j.1600-0447.2007.00998.x

[b3] Cuijpers P. & Smit F. Subthreshold depression as a risk indicator for major depressive disorder: a systematic review of prospective studies. Acta. Psychiat. Scand. 109, 325–331 (2004). 15049768 10.1111/j.1600-0447.2004.00301.x

[b4] Goldney R., Fisher L., Dal Grande E. & Taylor A. Subsyndromal depression: prevalence, use of health services and quality of life in an Australian population. Soc. Psych. Psych. Epid. 39, 293–298 (2004). 10.1007/s00127-004-0745-5 15085331

[b5] Bora E., Harrison B. J., Davey C. G., Yücel M. & Pantelis C. Meta-analysis of volumetric abnormalities in cortico-striatal-pallidal-thalamic circuits in major depressive disorder. Psychol. Med. 42, 671–681 (2012). 21910935 10.1017/S0033291711001668

[b6] Drevets W. C., Price J. L. & Furey M. L. Brain structural and functional abnormalities in mood disorders: implications for neurocircuitry models of depression. Brain. Struct. Funct. 213, 93–118 (2008). 18704495 10.1007/s00429-008-0189-x PMC2522333

[b7] Koolschijn P. C. M. P., van Haren N. E. M., Lensvelt-Mulders G. J. L. M., Hulshoff Pol H. E. & Kahn R. S. Brain volume abnormalities in major depressive disorder: A meta-analysis of magnetic resonance imaging studies. Hum. Brain. Mapp. 30, 3719–3735 (2009). 19441021 10.1002/hbm.20801 PMC6871089

[b8] Grieve S. M., Korgaonkar M. S., Koslow S. H., Gordon E. & Williams L. M. Widespread reductions in gray matter volume in depression. NeuroImage. Clin. 3, 332–339 (2013). 24273717 10.1016/j.nicl.2013.08.016 PMC3814952

[b9] Du M. *et al.* Brain grey matter volume alterations in late-life depression. J. Psychiat. Neurosci. 39, 397–406 (2014). 10.1503/jpn.130275 PMC4214874 24949867

[b10] Hulvershorn L., Cullen K. & Anand A. Toward dysfunctional connectivity: a review of neuroimaging findings in pediatric major depressive disorder. Brain. Imaging. Behav. 5, 307–328 (2011). 21901425 10.1007/s11682-011-9134-3 PMC3216118

[b11] Foland-Ross L. C., Hardin M. G. & Gotlib I. H. Neurobiological markers of familial risk for depression. Curr. Top. Behav. Neurosci. 14, 181–206 (2013). 22573472 10.1007/7854_2012_213 PMC11881774

[b12] Romanczuk-Seiferth N. *et al.* Larger amygdala volume in first-degree relatives of patients with major depression. NeuroImage. Clin. 5, 62–68 (2014). 25003028 10.1016/j.nicl.2014.05.015 PMC4081974

[b13] Saleh K. *et al.* Impact of family history and depression on amygdala volume. Psychiat. Res-Neuroim. 203, 24–30 (2012). 10.1016/j.pscychresns.2011.10.004 22867951

[b14] Webb C. A., Weber M., Mundy E. A. & Killgore W. D. Reduced gray matter volume in the anterior cingulate, orbitofrontal cortex and thalamus as a function of mild depressive symptoms: a voxel-based morphometric analysis. Psychol. Med. 44, 1–11 (2014). 25066703 10.1017/S0033291714000348 PMC4280261

[b15] Spalletta G., Piras F., Caltagirone C. & Fagioli S. Hippocampal multimodal structural changes and subclinical depression in healthy individuals. J. Affect. Disord. 152, 105–112 (2014). 23800444 10.1016/j.jad.2013.05.068

[b16] Hayakawa Y. K. *et al.* Structural brain abnormalities in women with subclinical depression, as revealed by voxel-based morphometry and diffusion tensor imaging. J. Affect. Disord. 144, 263–268 (2013). 23141669 10.1016/j.jad.2012.10.023

[b17] Peterson B. S. *et al.* Cortical thinning in persons at increased familial risk for major depression. Proc. Natl. Acad. Sci. U. S. A. 106, 6273–6278 (2009). 19329490 10.1073/pnas.0805311106 PMC2669378

[b18] Karsten J. *et al.* Psychiatric history and subthreshold symptoms as predictors of the occurrence of depressive or anxiety disorder within 2 years. Brit. J. Psychiat. 198, 206–212 (2011). 10.1192/bjp.bp.110.080572 21357879

[b19] Martinot M. P. *et al.* White-matter microstructure and gray-matter volumes in adolescents with subthreshold bipolar symptoms. Mol. Psychiatr. 19, 462–470 (2013). 10.1038/mp.2013.44 PMC3965837 23628983

[b20] Dotson V. M., Davatzikos C., Kraut M. A. & Resnick S. M. Depressive symptoms and brain volumes in older adults: a longitudinal magnetic resonance imaging study. J. Psychiat. Neurosci. 34, 367–375 (2009). PMC2732743 19721847

[b21] Southerland D., Casanueva C. E. & Ringeisen H. Young adult outcomes and mental health problems among transition age youth investigated for maltreatment during adolescence. Child. Youth. Serv. Rev. 31, 947–956 (2009).

[b22] Rebbeck T. R., Weber A. L., Spangler E. & Zeigler-Johnson C. M. What stresses men? predictors of perceived stress in a population-based multi-ethnic cross sectional cohort. BMC. Public. Health. 13, 1–9 (2013). 23388399 10.1186/1471-2458-13-113 PMC3627635

[b23] Gogtay N. *et al.* Dynamic mapping of human cortical development during childhood through early adulthood. Proc. Natl. Acad. Sci. U. S. A. 101, 8174–8179 (2004). 15148381 10.1073/pnas.0402680101 PMC419576

[b24] Lebel C. & Beaulieu C. Longitudinal development of human brain wiring continues from childhood into adulthood. J. Neurosci. 31, 10937–10947 (2011). 21795544 10.1523/JNEUROSCI.5302-10.2011 PMC6623097

[b25] Paus T., Keshavan M. & Giedd J. N. Why do many psychiatric disorders emerge during adolescence? Nat. Rev. Neurosci. 9, 947–957 (2008). 19002191 10.1038/nrn2513 PMC2762785

[b26] Hamilton J. P., Siemer M. & Gotlib I. H. Amygdala volume in major depressive disorder: a meta-analysis of magnetic resonance imaging studies. Mol. Psychiatr. 13, 993–1000 (2008). 10.1038/mp.2008.57 PMC2739676 18504424

[b27] Holmes A. J. *et al.* Individual differences in amygdala-medial prefrontal anatomy link negative affect, impaired social functioning, and polygenic depression risk. J. Neurosci. 32, 18087–18100 (2012). 23238724 10.1523/JNEUROSCI.2531-12.2012 PMC3674506

[b28] Gerritsen L. *et al.* Amygdala to hippocampal volume ratio is associated with negative memory bias in healthy subjects. Psychol. Med. 42, 335–343 (2012). 21740626 10.1017/S003329171100122X

[b29] Vincent J. L., Kahn I., Snyder A. Z., Raichle M. E. & Buckner R. L. Evidence for a frontoparietal control system revealed by intrinsic functional connectivity. J. Neurophysiol. 100, 3328–3342 (2008). 18799601 10.1152/jn.90355.2008 PMC2604839

[b30] Cabeza R., Ciaramelli E., Olson I. R. & Moscovitch M. The parietal cortex and episodic memory: an attentional account. Nat. Rev. Neurosci. 9, 613–625 (2008). 18641668 10.1038/nrn2459 PMC2692883

[b31] Müller V. I., Cieslik E. C., Laird A. R., Fox P. T. & Eickhoff S. B. Dysregulated left inferior parietal activity in schizophrenia and depression: functional connectivity and characterization. Front. Hum. Neurosci. 7, 268 (2013). 23781190 10.3389/fnhum.2013.00268 PMC3679482

[b32] Ries M. L., Wichmann A., Bendlin B. B. & Johnson S. C. Posterior cingulate and lateral parietal gray matter volume in older adults with depressive symptoms. Brain. Imaging. Behav. 3, 233–239 (2009). 19701486 10.1007/s11682-009-9065-4 PMC2728909

[b33] Qiu L. *et al.* Regional increases of cortical thickness in untreated, first-episode major depressive disorder. Transl. Psychiat. 4, e378 (2014). 10.1038/tp.2014.18 PMC4012282 24713859

[b34] Disner S. G., Beevers C. G., Haigh E. A. P. & Beck A. T. Neural mechanisms of the cognitive model of depression. Nat. Rev. Neurosci. 12, 467–477 (2011). 21731066 10.1038/nrn3027

[b35] Maddock R. J., Garrett A. S. & Buonocore M. H. Posterior cingulate cortex activation by emotional words: fMRI evidence from a valence decision task. Hum. Brain. Mapp. 18, 30–41 (2003). 12454910 10.1002/hbm.10075 PMC6871991

[b36] Adler C. M. *et al.* Voxel-based study of structural changes in first-episode patients with bipolar disorder. Biol. Psychiat. 61, 776–781 (2007). 17027928 10.1016/j.biopsych.2006.05.042

[b37] Soriano-Mas C. *et al.* Cross-sectional and longitudinal assessment of structural brain alterations in melancholic depression. Biol. Psychiat. 69, 318–325 (2011). 20875637 10.1016/j.biopsych.2010.07.029

[b38] Raichle M. E., MacLeod A. M., Snyder A. Z., Powers W. J., Gusnard D. A. & Shulman G. L. A default mode of brain function. Proc. Natl. Acad. Sci. U. S. A. 98, 676–682 (2001). 11209064 10.1073/pnas.98.2.676 PMC14647

[b39] Whitfield-Gabrieli S. & Ford J. M. Default mode network activity and connectivity in psychopathology. Annu. Rev. Clin. Psycho. 8, 49–76 (2012). 10.1146/annurev-clinpsy-032511-143049 22224834

[b40] Sheline Y. I., Price J. L., Yan Z. & Mintun M. A. Resting-state functional MRI in depression unmasks increased connectivity between networks via the dorsal nexus. Proc. Natl. Acad. Sci. U. S. A. 107, 11020–11025 (2010). 20534464 10.1073/pnas.1000446107 PMC2890754

[b41] Taki Y. *et al.* Male elderly subthreshold depression patients have smaller volume of medial part of prefrontal cortex and precentral gyrus compared with age-matched normal subjects: a voxel-based morphometry. J. Affect. Disord. 88, 313–320 (2005). 16150493 10.1016/j.jad.2005.08.003

[b42] Chen M. C., Hamilton J. P. & Gotlib I. H. Decreased hippocampal volume in healthy girls at risk of depression. Arch. Gen. Psychiat. 67, 270–276 (2010). 20194827 10.1001/archgenpsychiatry.2009.202 PMC2845291

[b43] Kessler R. C. *et al.* Lifetime and 12-month prevalence of DSM-III-R psychiatric disorders in the United States: results from the National Comorbidity Survey. Arch. Gen. Psychiat. 51, 8–9 (1994). 8279933 10.1001/archpsyc.1994.03950010008002

[b44] Oitzl M. S., Champagne D. L., van der Veen R. & De Kloet E. R. Brain development under stress: hypotheses of glucocorticoid actions revisited. Neurosci. Biobehav. Rev. 34, 853–866 (2010). 19631685 10.1016/j.neubiorev.2009.07.006

[b45] Beck A. T., Steer R. A. & Brown G. Manual for the Beck Depression Inventory-II (Psychological Corporation, Texas, 1996).

[b46] First M. B., Spitzer R. L., Gibbon M. & Williams J. B. W. Structured Clinical Interview for DSM-IV-TR Axis I Disorders, Research Version, Patient Edition. (SCID-I/P) (Biometrics Research, New York State Psychiatric Institute, New York, 2002).

[b47] Sun C., Wu Z., Wu Z. & Xu S. Age differences in RAVEN test and the relation between the differences and memory training of “method of loci”. Acta. Psychol. Sinica. 26, 59–63 (1994).

[b48] Jung R. E. & Haier R. J. The parieto-frontal integration theory (P-FIT) of intelligence: converging neuroimaging evidence. Behav. Brain. Sci. 30, 135–154 (2007). 17655784 10.1017/S0140525X07001185

[b49] Li H. & Sun J. *et al.* Neuroanatomical differences between men and women in help-seeking coping strategy. Sci. Rep. 4, 5700 (2014). 25027617 10.1038/srep05700 PMC4099976

[b50] Beck A. T., Steer R. A. & Carbin M. G. Psychometric properties of the Beck Depression Inventory: Twenty-five years of evaluation. Clin. Psychol. Rev. 8, 77–100 (1988).

[b51] Ashburner J. A fast diffeomorphic image registration algorithm. Neuroimage. 38, 95–113 (2007). 17761438 10.1016/j.neuroimage.2007.07.007

[b52] Mühlau M. *et al.* Structural brain changes in tinnitus. Cere. Cortex. 16, 1283–1288 (2006). 10.1093/cercor/bhj070 16280464

[b53] Li H. & Li W. *et al.* Examining brain structures associated with perceived stress in a large sample of young adults via voxel-based morphometry. Neuroimage. 92, 1–7 (2014). 24495811 10.1016/j.neuroimage.2014.01.044

[b54] Worsley K. *et al.* A unified statistical approach for determining significant signals in images of cerebral activation. Hum. Brain. Mapp. 4, 58–73 (1996). 20408186 10.1002/(SICI)1097-0193(1996)4:1<58::AID-HBM4>3.0.CO;2-O

[b55] Maldjian J. A., Laurienti P. J., Kraft R. A. & Burdette J. H. An automated method for neuroanatomic and cytoarchitectonic atlas-based interrogation of fMRI data sets. Neuroimage. 19, 1233–1239 (2003). 12880848 10.1016/s1053-8119(03)00169-1

[b56] Ebner N. C., Johnson M. K. & Fischer H. Neural mechanisms of reading facial emotions in young and older adults. Front. Psychol. 3, 223–223 (2012). 22798953 10.3389/fpsyg.2012.00223 PMC3394436

[b57] Hayasaka S., Phan K. L., Liberzon I., Worsley K. J. & Nichols T. E. Nonstationary cluster-size inference with random field and permutation methods. Neuroimage. 22, 676–687 (2004). 15193596 10.1016/j.neuroimage.2004.01.041

[b58] Takeuchi H. *et al.* A voxel-based morphometry study of gray and white matter correlates of a need for uniqueness. Neuroimage. 63, 1119–1126 (2012). 22926287 10.1016/j.neuroimage.2012.08.037

